# miR-100-5p Enhances Cell Cycle-Mediated Chemoresistance by Modulating the CTDSPL/pRB/E2F1 Signaling Pathway in Oxaliplatin-Resistant Colorectal Cancer Cells

**DOI:** 10.32604/or.2026.073080

**Published:** 2026-03-23

**Authors:** Yen-Pin Chen, Rathinasamy Baskaran, Hema Sri Devi, Chaouhan Hitesh Singh, Yu-Jung Lin, Marthandam Asokan Shibu, Wei-Wen Kuo, Shih-Chieh Liao, Ming-Cheng Chen, Tso-Fu Wang, Chi-Cheng Li, Tsung-Jung Ho, Tzu-Ching Shih, Shinn-Zong Lin, Chih-Yang Huang

**Affiliations:** 1 School of Medicine, National Defense Medical University, Taipei, Taiwan; 2 Department of Orthopedic Surgery, Tri-Service General Hospital, Taipei, Taiwan; 3 Department of Orthopedics, Taichung Armed Forces General Hospital, Taichung, Taiwan; 4 Cardiovascular and Mitochondrial Related Disease Research Center, Hualien Tzu Chi Hospital, Buddhist Tzu Chi Medical Foundation, Hualien, Taiwan; 5 Department of Research, Taipei Tzu Chi Hospital, Buddhist Tzu Chi Medical Foundation, New Taipei, Taiwan; 6 Department of Biotechnology, Bharathiar University, Coimbatore, India; 7 Department of Biological Science and Technology, China Medical University, Taichung, Taiwan; 8 Graduate Institute of Chinese Medical Science, China Medical University, 91 Hsueh-Shih Road, Taichung, Taiwan; 9 Department of Surgery, Division of Colorectal Surgery, Taichung Veterans General Hospital, Taichung, Taiwan; 10 Department of Hematology and Oncology, Hualien Tzu Chi Hospital, Buddhist Tzu Chi Medical Foundation, Hualien, Taiwan; 11 International Medical Center, Hualien Tzu Chi Hospital, Buddhist Tzu Chi Medical Foundation, Hualien, Taiwan; 12 Department of Chinese Medicine, Hualien Tzu Chi Hospital, Buddhist Tzu Chi Medical Foundation, Tzu Chi University, Hualien, Taiwan; 13 Department of Biomedical Imaging and Radiological Science, China Medical University, Taichung, Taiwan; 14 Buddhist Tzu Chi Foundation Hospital, Hualien, Taiwan; 15 Bioinnovation Center, Buddhist Tzu Chi Medical Foundation, Hualien, Taiwan; 16 Department of Neurosurgery, Hualien Tzu Chi Hospital, Buddhist Tzu Chi Medical Foundation, Hualien, Taiwan; 17 Graduate Institute of Biomedical Sciences, China Medical University, Taichung, Taiwan; 18 Department of Medical Laboratory Science and Biotechnology, Asia University, Taichung, Taiwan; 19 Department of Medical Research, China Medical University Hospital, China Medical University, Taichung, Taiwan

**Keywords:** miR-100-5p, C-terminal domain small phosphatase-like (CTDSPL)/retinoblastoma protein (pRB)/E2F transcription factor 1 (E2F1), chemoresistance, cell cycle progression, colorectal cancers

## Abstract

**Objective:**

MicroRNAs (miRNAs) are small, non-coding RNAs that play a key role in the development of chemoresistance in various cancer types, including colorectal cancer (CRC). In this study, we aimed to study the underlying mechanisms of miRNA in chemotherapy-resistant CRC.

**Methods:**

LoVo CRC cell line was exposed to oxaliplatin at an increased dose, and cells were cultured in the presence of oxaliplatin to develop LoVoOXR cells. Microarray and Quantitative Reverse Transcription Polymerase Chain Reaction (qRT-PCR), western blot, and transwell assay were used to evaluate the chemoresistance in LoVo^OXR^ CRC cells.

**Results:**

Microarray and qRT-PCR analysis showed an increased expression of miR-100-5p in LoVo^OXR^ cells. MTT assay and flow cytometry analysis revealed less apoptosis and higher cell viability in LoVo^OXR^ cells. mRNA prediction target gene analysis showed *C-terminal domain small phosphatase-like (CTDSPL)*, a phosphatase-like tumor suppressor, as a key target of miR-100-5p. CTDSPL expression was low in LoVo^OXR^ cells compared to LoVo^WT^ cells. miR-100-5p regulates G1/S and S-phase transitions and inhibits differentiation by targeting the CTDSPL/pRB/E2F1 signaling pathway, which involves the modulation of cell cycle effectors in LoVo^OXR^ cells. Further, we found that forkhead box P3 (FOXP3), as the upstream target of miR-100-5p, is highly expressed in LoVo^OXR^ cells. Inhibiting miR-100-5p and FOXP3 down-regulates miR-100-5p expression, while increased CTDSPL expression contributed to reduced cell proliferation and promoted cell apoptosis in LoVo^OXR^ CRC cells.

**Conclusions:**

miR-100-5p plays an oncogenic role in inducing chemoresistance through modulation of the CTDSPL/retinoblastoma protein (pRB)/E2F transcription factor 1 (E2F1) axis in CRC cells.

## 1 Introduction

According to the colorectal cancer statistic 2023 report, colorectal cancer is the third most commonly diagnosed cancer and the second most common cause of cancer death in the United States [1]. The American Cancer Society updates colorectal cancer statistics every three years using incidence data from population-based registries and death data from the National Center for Health Statistics. In spite of the ongoing decline in the overall number of deaths from colorectal cancer (CRC) in recent years, the diagnosis of CRC is increasingly being made at a younger age, in the left colon/rectum, and at a more advanced stage [1]. Despite advances in surgical techniques, chemotherapy, and radiotherapy, colorectal cancer remains the second leading cause of cancer-related deaths worldwide [2]. Clinical advances in colorectal cancer treatment, adjuvant systemic chemotherapy with cytotoxic drugs are recommended as the best palliative therapeutic approach for patients with advanced CRC, and are the first-line therapeutic option for patients with metastatic relapse and spread [3,4]. 5-fluorouracil (5-FU) in combination with oxaliplatin (L-OHP) or irinotecan (CPT-11) is generally more effective and has improved the 5-year survival rate for advanced CRC cancer patients by more than 65% [5]. In spite of advances in early detection and multimodal therapy, colorectal cancer recurrence remains a major clinical challenge, with approximately 20%–30% of patients developing relapse within five years after curative treatment, most commonly during the first 2–3 years of follow-up [1]. Most CRC patients eventually develop resistance to these chemotherapy agents in different mechanisms [6–8]. As a result, it is preferable to elucidate the underlying molecular mechanisms associated with chemoresistance because this knowledge may lead to the development of new strategies to overcome drug resistance in CRC patients who do not respond well to such treatments.

MicroRNAs (miRNAs) are endogenous RNA molecules that are small, evolutionarily conserved, single-stranded, non-coding RNA molecule (19–22 nucleotide) and regulate the expression of target genes by cleaving primary mRNA (pri-miRNA) or through base-pairing with target mRNA [9,10]. Numerous studies have discovered that miRNA plays an important role in a variety of biological processes in cancer, including cell proliferation, angiogenesis, invasion, and apoptosis [11,12] and is closely linked to the development of chemoresistance in cancer cells, particularly in colorectal cancer [13,14], which indicates miRNAs might be a potential target for cancer treatment. Previous research has suggested that miR-100-5p expression may play an oncogenic [15] or tumor suppressor [16] role in various types of cancer. miR-100 plays a crucial role in regulating tumor cell proliferation, migration, and invasion by targeting the 3^′^-UTR of its target genes in many cancer types [17]. miR-100 acts as a tumor suppressor in colorectal cancer by reducing leucine-rich repeat-containing G protein-coupled receptor 5 (LGR5) expression [18] and limits the cell proliferation, migration and invasion of breast cancer cells through the downregulation of Forkhead box A1 (FOXA1) [19]. Interestingly, some studies suggested that miR-100-5p could improve the anticancer effects of chemotherapeutic drugs in a variety of human cancers [20]. However, the precise molecular mechanisms by which the miR-100-5p gene regulates oxaliplatin resistance in CRC cells remain unknown.

Cell cycle-mediated chemoresistance, also known as cell cycle status, may alter tumor cell responses to chemotherapeutic agents. The *C-terminal domain small phosphatase-like* (*CTDSPL)* gene, formerly known as RBPS3 or HYA22, is a phosphatase-like tumor suppressor gene located at 3p21.3 that belongs to the small C-terminal domain phosphatase family and removes the phosphate group from Rb1 serine on ser-807 and ser-811 [21]. CTDSPL regulates the pRB/E2F1 signaling pathway and causes cell cycle arrest at the G1/S transition. miR-100-5p has been shown to regulate myeloid differentiation in both uveal melanoma and acute myeloid leukemia cells by targeting CTDSPL [22,23]. However, the role of CTDSPL in the regulation of cell growth in CRC cells is still unknown.

This study aimed to determine whether miR-100-5p contributes to oxaliplatin resistance in colorectal cancer and to investigate the underlying molecular mechanisms. We specifically hypothesized that miR-100-5p promotes chemoresistance by suppressing the CTDSPL and subsequently activating the pRB/E2F1 signaling pathway, thereby enhancing cell cycle progression and reducing oxaliplatin-induced apoptosis.

## 2 Materials and Methods

### 2.1 Cell Culture, Antibodies, and Reagents

LoVo CRC cell line was obtained from the Bioresource Collection and Research Center (BCRC number: 60148), (Hsinchu, Taiwan). LoVo cell line is a type of human colon adenocarcinoma isolated from large intestine of a 56-year-old Caucasian male with grade IV Dukes C colorectal cancer. According to the supplier’s documentation, this line has been short tandem repeat (STR) authenticated and verified for species and identity. The cell line was tested negative for mycoplasma contamination prior to shipment. In our laboratory, cells were routinely screened for mycoplasma using mycoplasma assay kit (ab289834) (Abcam, Cambridge, UK) and maintained under sterile conditions. LoVo CRC cells were maintained in Dulbecco’s Modified Eagle’s Medium (DMEM)-Low glucose (D5523-1L, Sigma-Aldrich, St. Louis, MO, USA) supplemented with 10% Fetal bovine serum (FBS) (F0926, Sigma-Aldrich, St. Louis, MO, USA), 1% penicillin/streptomycin, and sodium bicarbonate at 37°C, with 5% CO_2_ and 80%–90% humidified condition. The cell medium was changed after 48 h of subculture. Oxaliplatin powder and 1-(4,5-dimethylthiazol-2-yl)-3,5-diphenylformazan (MTT) (M6494, Thermo Fisher Scientific, Waltham, MA, USA). CTDSPL (NBP1-91814) (Novus Biologicals Co., Centennial, CO, USA); E2F1 (#3742), phosphorylated RB (#9308), and RB (#9309), p53 (#2524), phosphorylated-p53 (#9284) (Cell Signaling Technology, Inc. Beverly, MA, USA); cyclin E1 (ab33911), cyclin A1 (ab270940) (Abcam, Cambridge, UK); and E-cadherin (sc-8426), Vimentin (sc-32322), α-Tubulin (sc-5286), β-Catenin (sc-7963), Cdk4 (sc-23896), Cdk6 (sc-7961), Cdc25a (sc-7389), cyclin D1 (sc-246), cyclin B1 (sc-752), cyclin A1, PCNA (sc-56), ERK1/2 (sc-514032), phosphorylated-ERK1/2 (sc-7383), AKT (sc-5298), phosphorylated-AKT (sc-514032), p19 (sc-166774), p27 (sc-1641), p15/16 (sc-377412), Ki67 (sc-23900) and GAPDH (sc-32233) (Santa Cruz Biotechnology, Inc. Santa Cruz, CA, USA). Secondary antibodies used were HRP conjugated-goat anti-mouse (sc-516102) and anti-rabbit IgG (sc-2357) antibodies: these were purchased from Santa Cruz Biotechnology, Inc. (Dallas, TX, USA). All antibodies were used in this study.

### 2.2 Viability Assay

The MTT method was used following the procedure of Chang et al. [24] with minor modifications. LoVo^WT^ and LoVo^OXR^ cells (5 × 10^4^ cells/well) were cultivated in triplicate in DMEM-Low Glucose with 10% FBS in 24 well plates for 24 h. The cells were treated with oxaliplatin at concentration (0, 5, 10, 15, 20, 25, and 30 µg/mL) and incubated for 24 h. The treated medium was replaced with 100 µL MTT solution (15 mg/50 mL) then incubated at 37 °C for 2–5 h until purple precipitate forms. The supernatant was discarded, and 150 µL of DMSO per well was added to dissolve the blue MTT formazan crystals. The absorbance was measured at 570 nm using a multiwall Enzyme-Linked Immunosorbent Assay (ELISA) plate reader (SpectraMax mini, Molecular Devices, Palo Alto, CA, USA). IC_50_ was taken as the cell population’s concentration of 50% death.

Cell survival (percentage) = [1 − (OD experiment sample/ODcontrol sample)] × 100%. (*n* = 3)

### 2.3 Establishment of Oxaliplatin-Resistant LoVo Cancer Cells

Parental LoVo cells were first assayed for oxaliplatin sensitivity by MTT by treating it for 24 h. The IC_50_ (1) was found to be 15 μM. This dose range (0–25 μM) was used to guide the resistance-selection protocol. After drug treatment live cells were returned to drug-free medium and grown to ∼70%–80% confluence (typically several days) before the next treatment. Surviving cells were again washed and expanded. In the second cycle, MTT assay on this survivor population established a new IC_50_ the IC_50_ (2), which was found to be 20 μM. The resulting population was finally maintained in 20 μM oxaliplatin (above the previous IC_50_). The same cycle is repeated to another new IC_50_ (3) at 25 μM. At this stage we designated the line as LoVoOXR. Under these conditions the oxaliplatin IC_50_ of LoVoOXR increased to >45 μM (∼4-times higher than parental). Finally, we note that the resistant phenotype was stable after prolonged culture without oxaliplatin.

### 2.4 Microarray Analysis

miRNA Microarray Services (Service Code: 2h213102401; Human miRNA OneArray^®^ V6.1) were used to analyze the microRNA expression profile. Following the manufacturer’s instructions, total RNA was isolated from the LoVo^WT^ and LoVo^OXR^ cell lines using an RNA MiniPrep kit (R1055) (Zymo Research Corporation, Irvine, CA, USA). RNA purity was quantified by NanoDrop^TM^ One/One^C^ Spectrophotometer (Thermo Scientific, USA), and all the samples with A260/280 ratios between 1.95–2.05 were used and 1 µg of RNA per sample was used for microarray analysis. Raw microarray data were preprocessed using the OneArray^®^ Expression Analysis software. Background subtraction and global normalization were applied. Differential expression analysis was performed using the manufacturer-recommended workflow and validated through R-based statistical analysis. miRNAs with a fold change ≥ 2.0 (log_2_ FC ≥ 1.0) and *p* < 0.05 were considered significantly differentially expressed. The fold change was calculated by comparing the expression levels of various miRNAs in LoVo^OXR^ to those in LoVo^WT^ in log_2_ format.

### 2.5 mRNA Prediction and Analysis

Three common miRNAs predict target and binding sites in publicly available algorithms: http://www.microrna.org for the Miranda algorithm, http://www.targetscan.org for the TargetScan algorithm, and http://mirtar.mbc.nctu.edu.tw/human/for the miRTar base algorithm. All were used to predict the putative targets of miR-100-5p. Integrated function and pathway analysis were performed using DAVID bioinformatics resources (V6.8) (http://david.abcc.ncifcrf.gov/), accessed on 24 March 2025. Enrichment analysis was conducted using default background settings, and significant terms were defined by *p* < 0.05 and gene count ≥ 2. Differentially expressed genes were selected using a log_2_ fold-change cutoff ≥ 1.0 and *p* < 0.05. Cluster analysis of enriched pathways and gene sets was performed using hierarchical clustering with Euclidean distance to group functionally related targets.

### 2.6 RNA Isolation and Quantitative Reverse Transcription-Polymerase Chain Reaction (qRT-PCR) Assay

Total RNA was extracted from LoVo^WT^ and LoVo^OXR^ using GeneJET RNA purification (Thermo Fisher Scientific, Waltham, MA, USA) and reverse transcribed to cDNA using a GScript First-Strand Synthesis Kit (MB305-0050) (cDNA synthesis kit). Real-Time PCR (QuantStudio™ 5, Thermo Fisher Scientific, Waltham, MA, USA), and ORA™ SEE qPCR Green ROX L Mix, 2X (HighQu professionally simple, USA) with a total reaction volume of 20 µL containing 10 µL ORA™ SEE qPCR Mix, 300 nM (each) forward and reverse primers and 500 ng cDNA were used. The cycling conditions were, 95°C for 2 min, followed by 40 cycles of 95°C for 15 s, 60°C for 30 s, and 72°C for 30 s, with a melt-curve analysis from 65°C to 95°C to confirm specificity. The primers for miR-100-5p; forward: AACCCGUAGAUCCGAACUUGUG, mRQ 3^′^ universal reverse primer (10 µM); TGGTGTCGTGGAGTCG, U6 Forward Primer (10 µM); CTCGCTTCGGCAGCACA, and U6 Reverse Primer (10 µM); AACGCTTCACGAATTTGCGT were obtained from the Mir-X miRNA First-Strand Synthesis Kit (638313, Takara Bio USA, Mountain View, CA, USA) and SYBR qRT-PCR user manual. U6 was used as an endogenous control. The gene expression is determined by the cyclic threshold Eq. (2)^−∆∆Ct^, where ∆Ct = (Ct microRNA − Ct U6 rRNA). Every reaction was performed in triplicate. The primers for the upstream target were *GATA binding factor 1 (GATA-1)*; forward: 5^′^-CAC TGA GCT TGC CAC ATC C-3^′^, reverse: 5^′^-ATG GAG CCT CTG GGG ATT A-3^′^, *CCAAT/enhancer-binding protein beta* (*CEBP-b)*; forward: 5^′^-CGC TTA CCT CGG CTA CCA-3^′^, reverse: 5^′^-ACG AGG AGG ACG TGG AGA G-3^′^, and *forkhead box P3* (*FOXP3)*; forward: 5^′^-CTT CCT TGA ACC CCA TGC-3^′^, reverse: 5^′^-GGA GGA GTG CCT GTA AGT GG-3^′^. Primer the downstream target was *C-terminal domain small phosphatase-like* (*CTDSPL)*; forward: 5^′^-AAC CCC AAG GAG GAC GAG-3^′^, reverse: 5^′^-CAG AAG AAG GAG CTA AGG ATG C-3^′^. *GAPDH*; forward: 5^′^-GCA CCG TCA AGG CTG AGA AC-3^′^, reverse: 5^′^-ATG GTG GTG AAG ACG CCA GT-3^′^ was used as an endogenous control.

### 2.7 Transfection

Over expression of miR-100-5p (mimic): AACCCGUAGAUCCGAACUUGUG, knockdown (inhibitor): CACAAGUUCGGAUCUACGGGTU, scrambled miRNA (mimic NC: UUCUCCGAACGUGUCACGUTT and inhibitor NC: CAGUACUUUUGUGUAGUACAA), (GeneDireX Inc., Las Vegas, NV, USA) and sh-RNA; sh-*CTDSPL* (sc-78502), sh-*FOXP3* (sc-43569), sh-*CEBP-b* (sc-44251) and sh-*GATA-1* (sc-29330) (Santa Cruz Biotechnology, Inc., Santa Cruz, CA, USA) were used to transfect the cells. LoVo^WT^ and LoVo^OXR^ cells were seeded at a density of 2 × 10^5^ cells per well in 6 well plates with confluency of 60%–80%. Transfection was accomplished using the jetPRIME^®^ (101000027, Polyplus Transfection Inc., Illkirch, France) Kit; For each well, 200 ng of miRNA mimic or inhibitor or 500 ng of shRNA plasmid was diluted in 200 µL of jetPRIME^®^ buffer, followed by the addition of 4 µL of jetPRIME^®^ reagent. The mixture was incubated for 10 min at room temperature and then added dropwise to the cells. Transfected cells were incubated in 5% CO_2_ at 37°C humidified condition for 24 h before being used in subsequent experiments.

### 2.8 Protein Quantification

Proteins were extracted using the Bradford reagent (#5000006, BIO-RAD, Hercules, CA, USA) and protein estimation was calculated using bovine serum albumin (BSA) (A7906-100G, Sigma-Aldrich, St. Louis, MO, USA) as a standard (2.0 mg/mL) according to the manufacturer’s instruction. The calculation standard curve is made from diluted BSA (0.5 mg/mL final concentration) at various protein concentrations (0.0, 0.1, 0.2, 0.3, 0.4 and 0.5 mg/mL). In a 96-well plate, 198 µL Bradford reagent was combined with 2.0 µL protein samples. Following that, we used a multi-well ELISA plate reader (SpectraMax Mini, Molecular Devices, Palo Alto, CA, USA) to measure the optical density of the sample at 595 nm and calculated the protein amounts using BSA standard curve.

### 2.9 Western Blotting

We examined the protein expression following the method [25] with minor modifications. The protein was centrifuged at 8000× *g* for 15–20 min at 4°C, and 45.0 µg of protein was boiled (95°C, 10 min) with an appropriate 5× sample buffer [10% SDS, 50% glycerol, 0.1% bromophenol blue, 250 mM Tris-HCl (pH-6.8) and 5% β-mercaptoethanol], and then loaded on 10%–12% sodium dodecyl sulfate-polyacrylamide gels for (SDS-PAGE) at 80–100 V for 2 h. Next, we transferred proteins onto a polyvinylidene difluoride membrane (PVDF) (IPVH85R, Millipore Corporation Limited, Bedford, MA, USA) in transfer buffer (25 mM Tris base, 190 mM glycine, and 20% methanol) using a BIO-RAD transfer system (Bio-Rad Laboratories, Munich, Germany). We blocked the protein in the membrane for one hour at room temperature (RT) with blocking buffer [5% (w/v) BSA, 1× transfer buffer saline, and 0.1% Tween-20]. We used primary antibodies (1:800 dilution for antibodies purchased from Santa Cruz, 1:1000 dilution for other antibodies in 1× TBST) at 4°C overnight. After that, we washed the membrane three times with 1× TBST and incubated with secondary antibodies (1:4500) at room temperature for 1 h anti-rabbit and anti-goat HRP-secondary antibodies. The protein signal was detected on an Image Bright i500 imaging system (Invitrogen™ iBright™ Imaging system, Thermo Fisher Scientific, Waltham, MA, USA), using Immobilon^®^Western chemiluminescent reagent (WBKLS0500, Millipore Corporation Limited, Bedford, MA, USA). The densitometry was analyzed using the ImageJ program 1.50 (National Institutes of Health, Bethesda, MD, USA). GAPDH and HDAC1 were used as endogenous control for normalization protein.

### 2.10 Immunocytochemistry Assay

The cells LoVo^WT^ and LoVo^OXR^ were cultured at a density 5 × 10^3^ cells per well of 8-well chamber slides and incubated for 24 h. Followed by fixation with 4% formaldehyde for 30–45 min at RT and then washed twice with 1× PBS. Permeabilization in 0.1% PBST (0.1% TRITON X-100 in 1× PBS solution) for 15 min at RT. Cells were blocked in blocking solution [4% (w/v) BSA + 0.1% Tween-20 in PBS] for 1–2 h at RT and incubated overnight in primary antibodies (same as 2.9) diluted with blocking solution at 4°C, washed three times for 5 min with 0.1% PBST. Followed by incubated secondary antibodies that were diluted in the ratio of 1:500 for the fluorophore Alexa 488 at room temperature for 1–2 h; these were finally washed three times for 5 min with 0.1% PBST. Coverslips were washed in distilled water before being mounted on a slide with a mounting medium DAPI-Aqueous (ab104139, Abcam, Cambridge, UK). Images were captured using a fluorescence microscope (Olympus Corporation, Model BX53, Olympus, Tokyo, Japan).

### 2.11 Quantitative Determination of Apoptosis: Annexin-V/PI Dual Staining Assay

A quantitative assessment of apoptosis was performed using fluorescein isothiocyanate (FITC) Annexin V which is based on the detection of phosphatidylserines. Briefly, LoVo^WT^ and LoVo^OXR^ cells were seeded in 6-well plates at 1 × 10^5^ cells per well for 24 h in DMEM. Then, the cells were treated with increasing concentration of oxaliplatin (0, 5.0, 10.0, 15.0, 20.0, and 25.0 μg/mL) for next 24 h. To analyze the Annexin-V/PI apoptosis assay, cells were collected, washed twice with ice-cold 1× PBS, suspended in 100 µL 1× binding buffer solution (25 mM CaCl_2_, 1.4 M NaCl, and 0.1 M Hepes/NaOH, pH 7.4), and stained with 5 µL of FITC-conjugated Annexin V (Annexin V-FITC) and 5 µL of propidium iodide (PI) followed by incubated in the dark for at least 15 min. The stained cells were diluted by the addition of 400 µL binding buffer, and the cells (10,000 cells per sample) were immediately analyzed by flow-cytometry [FACS Canto^TM^ system (BD Biosciences, Waltham, MA, USA)] at the Fluorescence Activated Cell Sorting (FACS) Core Facility, Tzu-Chi Buddhist General Hospital, Taiwan. Annexin V–FITC was detected using 488 nm excitation with 530/30 nm emission, and propidium iodide was detected using 488 nm excitation with 585/42 nm emission. The data were evaluated by CellQuest (version 5.0; BD Biosciences), and the apoptosis rate was analyzed by ModFIT software (Mac V1.01 software, Verity Software House, Topsham, ME, USA). The cell death was calculated as 100% [1 − (Q3Drug/Q3Control)].

### 2.12 Transwell Migration and Invasion Assay

A total of 5 × 10^4^ treatment cells were resuspended in 200 μL of serum-free DMEM (D5523-1L, Sigma-Aldrich, St. Louis, MO, USA) and loaded in the upper compartment of a transwell chamber (Corning, New York, NY, USA; 24-well insert, pore size: 8 μM). The lower chamber was filled with 100 μL DMEM with 10% FBS (F0926, Sigma-Aldrich, St. Louis, MO, USA) as a chemoattractant. The cells were incubated for 48 h for migration assays and 72 h for invasion assays. For the invasion assay, the inserts were pre-coated with extracellular matrix gel Corning^®^ Matrigel^®^ Basement Membrane Matrix (CLS354234, BD Bioscience, Sparks, MD, USA). Matrigel was diluted to a 1:8 concentration (v/v) in serum-free DMEM, and 50 μL of the diluted Matrigel solution was added to each insert. At the end of each experiment, the cells on the upper surface of the membrane were removed, and the lower surface were fixed with 4% paraformaldehyde for 15 min at room temperature and stained with Giemsa for 20 min, rinsed with distilled water, and air dried. The number of cells in 5 randomly selected areas was selected under microscopic fields (×200) and counted. Invasion and migration rates were calculated as follows: Invasion/migration rate (%) = (average transmembrane cell number of the treatment group/average transmembrane cell number of the control group (no treatment)) 100%.

### 2.13 TCGA Expression and Survival Analysis

Publicly available colorectal cancer datasets from The Cancer Genome Atlas (TCGA-COAD) were analyzed to assess correlations among miR-100-5p, FOXP3, and CTDSPL expression. Normal (n = 120) and tumor (n = 480) tissue datasets were downloaded through the TCGA data portal (https://portal.gdc.cancer.gov/). Expression values (FPKM) were log-transformed and analyzed using R (v4.2.0). Pearson correlation coefficients were calculated to determine gene–gene associations. For survival analysis, patients were divided into “high” and “low” expression groups using the median expression value as the cutoff. Kaplan–Meier curves were generated using the survival and survminer R packages. Statistical significance was assessed using the log-rank test, and *p*-values were adjusted for multiple testing using the Benjamini–Hochberg false discovery rate (FDR) correction.

### 2.14 Transcription Factor Binding Site Prediction

Potential upstream transcription factors regulating miR-100-5p were predicted using the PROMO (v3.0.2) and TFBIND online tools (http://alggen.lsi.upc.es). The promoter region of miR-100-5p (−2 kb upstream) was analyzed using a 15% dissimilarity threshold. PROMO identified putative transcription factor binding motifs, while TFBIND cross-referenced sequences against the TRANSFAC database to generate binding positions, strand information, and dissimilarity scores.

### 2.15 Statistical Analyses

The data were analyzed in three independent biological triplicates, and the values are represented as the mean ± standard deviation. SigmaPlot version 11.0 (Systat Software Inc., San Jose, CA, USA) and GraphPad Prism software 7.0 (GraphPad Software, San Diego, CA, USA) were used to obtain the mean value data. A one-way analysis of variance was used to analyze normally distributed data. Followed by a Student *t*-test with a probability of less than 0.05% (*p* < 0.05). All values were considered representative and statistically significant.

## 3 Results

### 3.1 Establishment of an Oxaliplatin-Resistant CRC Cell Line

**Figure 1:**
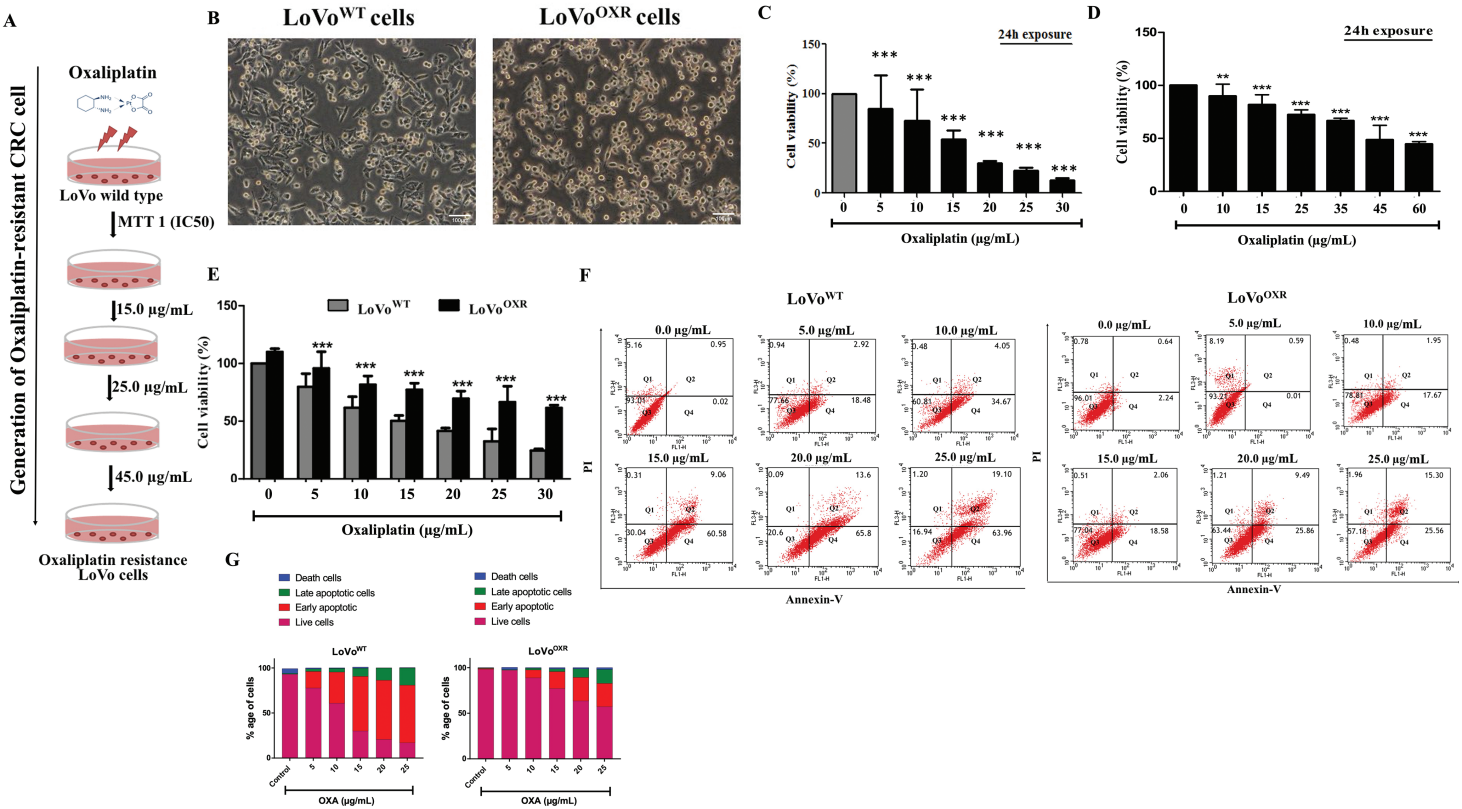
Generation of the stable drug-resistance colorectal cancer (CRC) cell line. (**A**) Generation of stable drug resistance cell lines through repeated subcultures with increased concentrations of oxaliplatin (15, 25, 35 and 45 µg/mL) was maintained over 6 months. (**B**) Images showing the morphology of parental and oxaliplatin-resistant LoVo cells (20× magnification). (**C**) The cell viability under oxaliplatin treatment was determined by treating parental LoVo cells in a dose dependent manner. (**D**) The 3-(4,5-dimethylthiazol-2-yl)-2,5-diphenyltetrazolium bromide (MTT) assay was used to determine oxaliplatin-resistant LoVo cells viability when challenged with an oxaliplatin IC_50_ dose. Resistance to oxaliplatin was increased compared to the control (**E**) Both parental cell and oxaliplatin-resistant cells were treated with varying concentration of oxaliplatin for 24 h and analysed for cell viability. (**F**) LoVo^WT^ and LoVo^OXR^ CRC cells exposed oxaliplatin challenge were incubated with annexin V and propidium iodide (PI) reagent, and flow-cytometry analysis was performed to measure the apoptotic cells death (early and late). (**G**). The data are presented as cell survival (fold change) and are presented as mean ± SD (*n* = 3). Significance ascribed as **p < 0.01, ***p < 0.001 vs. control.

In the present study, we generated stable oxaliplatin-resistant CRC cells (LoVo^OXR^) by plating 1 × 10^6^ LoVo cells in 10 cm plates with IC_50_ of oxaliplatin for 24 h, which resulted in 50% cell death. The procedure was repeated until the LoVo^OXR^ had at least a 3-fold greater IC_50_ (45 μg/mL) to oxaliplatin resistance than the LoVo parental (LoVo^WT^) CRC cell (Fig. 1A). Next, we confirmed that the morphological feature differs between LoVo^WT^ and LoVo^OXR^ cells. Based on microscopic observation, the morphological feature differences between LoVo^WT^ and LoVo^OXR^ are: LoVo^WT^ is more spindle-shaped than LoVo^OXR^, which has a polygonal or columnar shape (Fig. 1B). MTT assay results showing the cell viability of oxaliplatin-treated LoVo^WT^ CRC cells in a dose-dependent manner (Fig. 1C). LoVo cells were challenged with an oxaliplatin IC_50_ dose, and resistance to oxaliplatin was increased compared with the control. The resistance characteristics of LoVo^OXR^ cells were investigated using an MTT assay (Fig. 1D). To compare resistance between parental cells and resistant cells, both cells were treated with oxaliplatin in different dose from 5 to 30 μg/mL for 24 h. Cell viability was significantly decreased in the parental cell in a dose-dependent manner. However, no significant decrease in cell viability at lower concentrations was observed in oxaliplatin-resistant LoVo cells. Both cell lines demonstrated significantly different viability (Fig. 1E). Further, we investigated whether drug-induced apoptosis had an effect on the observed oxaliplatin-resistant LoVo cells. We performed a flow cytometry-based apoptosis assay, and the results showed that the apoptotic cell (early apoptotic and late apoptotic cells) rate was significantly increased in a dose-dependent manner in both parental and LoVo^OXR^ cells after 24 h of oxaliplatin exposure, whereas the cell death rate in LoVo^OXR^ cells was significantly lowered as compared to LoVo^WT^ CRC cell. The percentage of apoptotic cells in non-treated cells was nearly the same as in LoVo^WT^ and LoVo^OXR^ cells (Fig. 1F,G). Based on our findings, we classified our resistant cells as oxaliplatin-resistant in general.

### 3.2 Evaluation of the Cell Properties of Parental and Oxaliplatin-Resistant LoVo CRC Cell Lines

**Figure 2:**
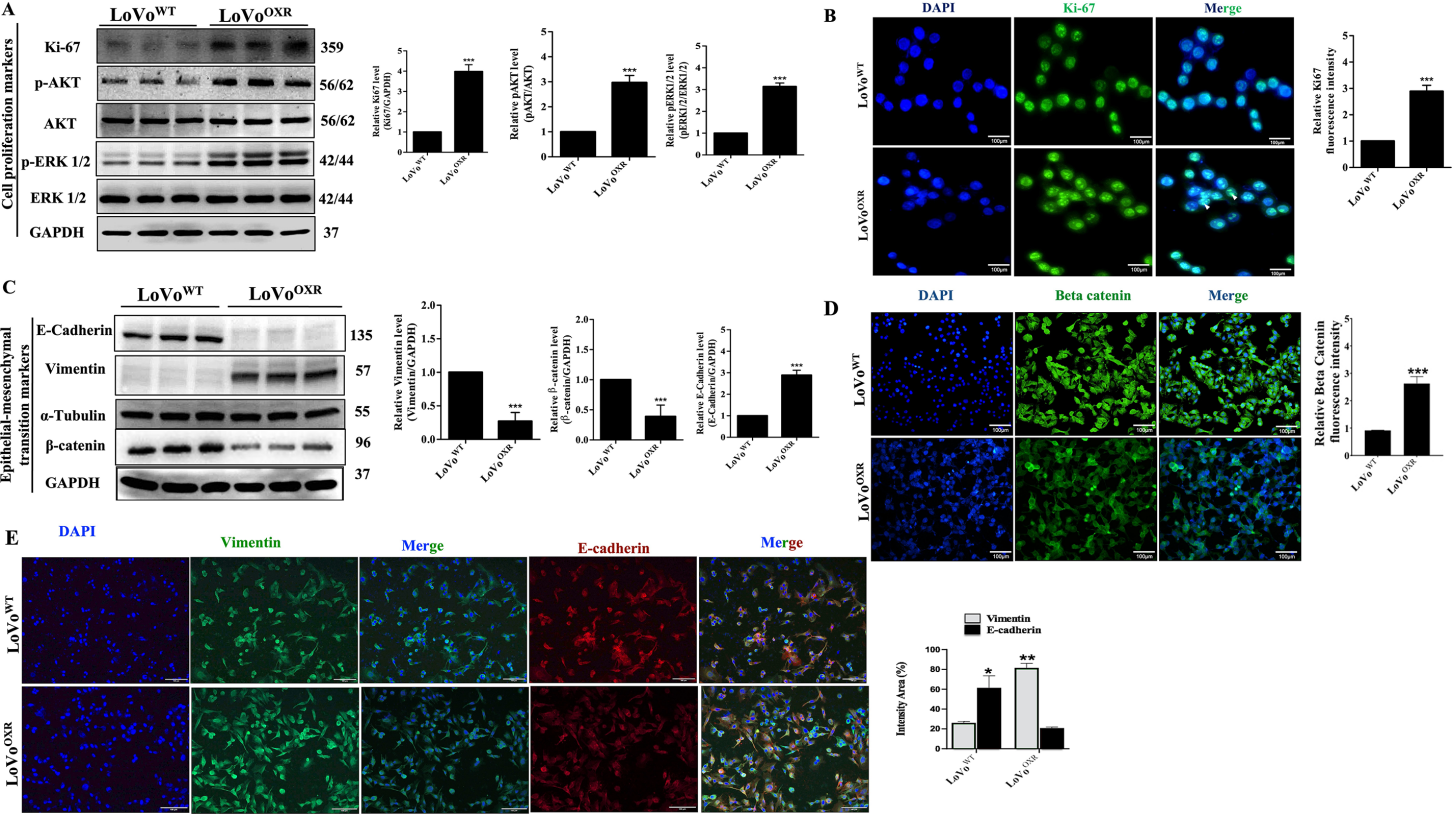
Cell properties altered between parental and oxaliplatin resistant (OXR)-LoVo cells. (**A**) Western blotting blotting analysis of the expression levels of the cell proliferation marker (Kiel-67 (Ki67), p-Protein Kinase B (AKT) and p-Extracellular signal-Regulated Kinase (ERK)1/2) in parental and oxaliplatin-resistant LoVo cells. (**B**) The immunofluorescence images showed that the expression level of Ki67 was greater in more cells of chemoresistance cells than in the parental cells. (**C**) Western blotting detected E-cadherin, Vimentin, α-tubulin and β-catenin Epithelial-Mesenchymal Transition (EMT) marker-related in oxaliplatin-resistant cells and parental cells. (**D**) The immunofluorescence images showed that the intensity of Vimentin and E-cadherin in the LoVo^OXR^ and LoVo^WT^ CRC cells. (**E**) Immunofluorescence images showing the expression of EMT markers vimentin and E-cadherin in the LoVo^OXR^ and LoVo^WT^ CRC cells. All protein expression normalized against GAPDH was used as a loading control. Quantification of the data was carried out by ImageJ software 1.50 and is presented as the mean ± SD (*n* = 3). Significance ascribed as *p < 0.05, **p < 0.01, ***p < 0.001 vs. oxaliplatin-resistant LoVo cells.

Following the result, we used western blot and immunofluorescence analyses to determine the effect of chemoresistance on cell properties such as cell proliferation and Epithelial-Mesenchymal Transition (EMT) markers in LoVo^WT^ and LoVo^OXR^ CRC cells. The result shows that the cell proliferation expression levels were related to proteins Ki67 (a primary proliferation marker of CRC cells), pAKT, and p-ERK1/2; those proteins were significantly higher in LoVo^OXR^ than in LoVo^WT^ CRC cell (Fig. 2A). Immunofluorescence result also shows that expression of Ki67 protein was significantly greater in chemoresistance cells than in the parental cells (Fig. 2B). Further, we examined the expression of EMT marker-related proteins E-cadherin, vimentin, and β-catenin in the parental and chemoresistance cells. The EMT marker-related protein showed that vimentin protein was higher in CRC cell lines, while E-cadherin protein was lower in LoVo^OXR^ than in LoVo^WT^ CRC cells (Fig. 2C). In addition, immunofluorescence results confirmed that the expression of beta-catenin protein was higher in LoVo^OXR^ than in LoVo^WT^ CRC cells (Fig. 2D). Furthermore, fluorescence intensity of EMT markers showed that in the LoVo^WT^, E-cadherin level was higher in LoVo^WT^ than in LoVo^OXR^, while vimentin level was higher in the LoVo^OXR^ compared to the LoVo^WT^ CRC cells (Fig. 2E).

### 3.3 Oxaliplatin-Resistant CRC Cell Line Express Increased Levels of miR-100-5p

**Figure 3:**
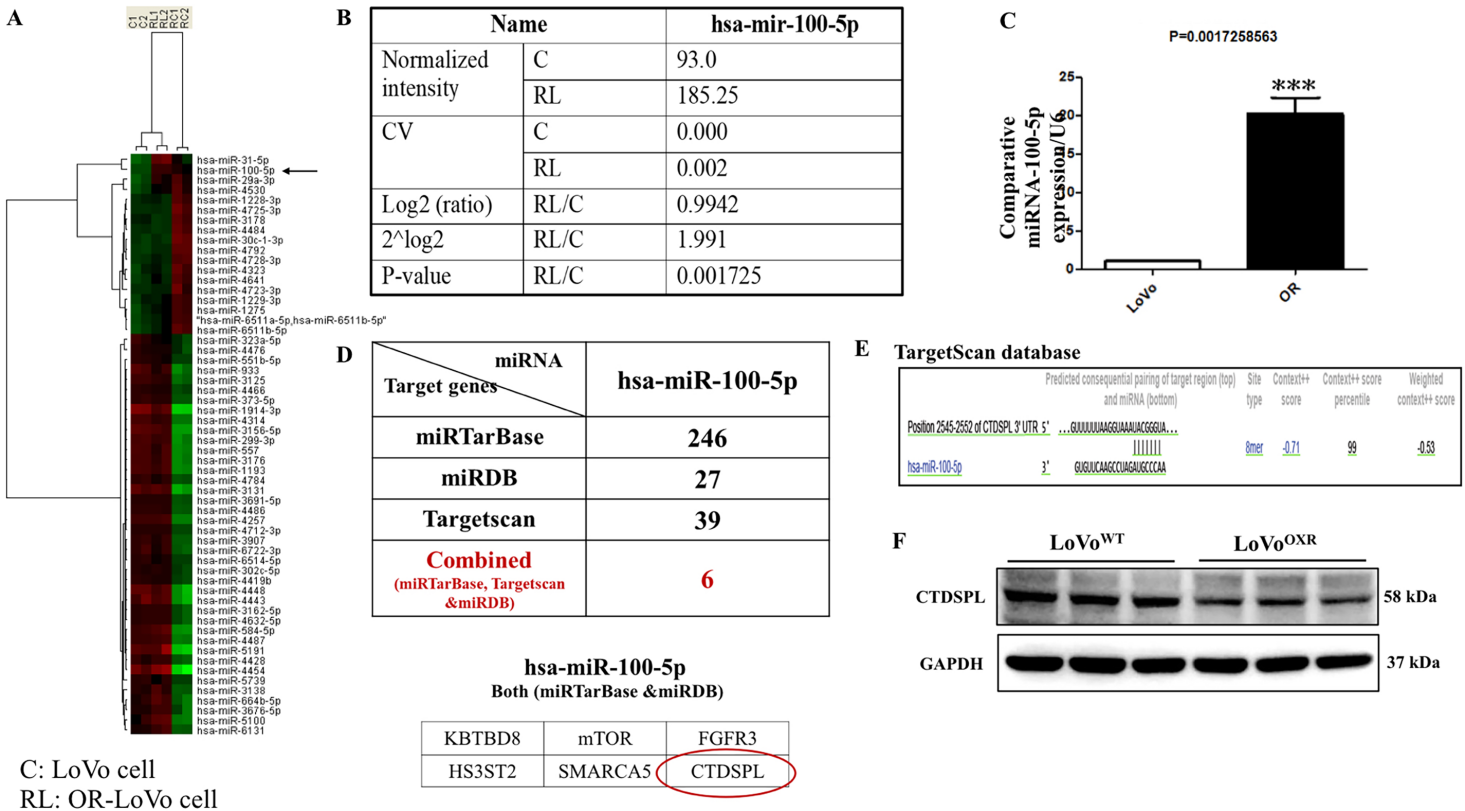
Oxaliplatin-resistant colorectal cancer cells highly express miR-100-5p. (**A**) Different miRNA expression levels in parental and oxaliplatin-resistant LoVo cells were examined by microarray analysis, wherein the red bar indicated up-regulated expression while the green bar indicated down-regulated expression. hsa-miR-100-5p was shown in the black arrow. (**B**) Table showing the detailed microarray analysis of hsa-miR-100-5p (C: parental LoVo cells and RL/C: oxaliplatin-resistant LoVo cells). (**C**) The graph shows the expression of hsa-miR-100-5p in parental and oxaliplatin resistant LoVo cells by the quantitative reverse transcription polymerase chain reaction (qRT-PCR) analysis. (**D**) We used three computational algorithms (miRTarBase, miRDB, and TargetScan Human) databases to predict the putative targets of hsa-miR-100-5p, and found 6 common predicted targets from the above-mentioned three online databases, including C-terminal domain small phosphatase-like (CTDSPL) (red circle). (**E**) Image showing the putative target binding sites of C-terminal domain small phosphatase-like (CTDSPL) to miR-100-5p. (**F**) Western blot analysis was used to confirm the expression level of CTDSPL in the two cell lines. All protein expression normalized against GAPDH was used as a loading control. Quantification of data was carried out by ImageJ software 1.50 and is presented as the mean ± SD (*n* = 3). Significance ascribed as ***p < 0.05 vs. oxaliplatin-resistant LoVo cells.

To screen the potential role of miRNA targets in CRC chemoresistance, we analyzed the miRNA expression profile in oxaliplatin drug-resistant cell lines. The microarray analysis results showed that the aberrant expression levels of various miRNAs were different between LoVo^OXR^ and LoVo^WT^ cells. We identified a single miRNA with a high expression level, is miR-100-5p in LoVo^OXR^ cells than in LoVo^WT^ cells (Fig. 3A). The raw data of hsa-miR-100-5p expression between the two CRC cell lines shows that RL/C (C is LoVo cells; RL is OXR-LoVo cells) had a log2 value of 1.515 ≥ 0.8, 2^log2^ value of 2.85, and a *p*-value of 0.009647 < 0.05 (Fig. 3B). Furthermore, we verified the microarray data of miR-100-5p expression using qRT-PCR assay, and the results confirmed that miRNA-100-5p expression was highly increased in LoVo^OXR^ cells compared to that in LoVo^WT^ cells (Fig. 3C). These results revealed that aberrant miRNA expression levels in resistant cells might be associated with resistance to chemotherapy and miR-100-5p expression, which has an essential role in the chemoresistance development of LoVo cells to oxaliplatin. To ascertain the putative target genes of miR-100-5p, we screened the target genes of miR-100-5p using different types of bioinformatics-based miRNA target prediction databases are TargetScan (http://www.targetscan.org/), Miranda (http://www.microrna.org/) and mirTar (http://mirtar.mbc.nctu.edu.tw/human/) (Fig. 3D). Further to check the miR-100-5p expression correlation in CRC clinical samples we analyze TGCA dataset (Supplementary Fig. S1). Gene correlation analysis of miR-100-5p, FOXP3, and CTDSPL expression in normal (120 samples) and colon cancer patient’s sample (480 samples), in which we found negative correlation (r = −0.065), which validates our finding upregulation of miR-100-5p downregulates CTDSPL expression. Also, significant increased miR-100-5p expression and decreased CTDSPL expression in CRC patient’s vs. normal. Kaplan−Meier survival analysis showing expression levels of miR-100-5p had significantly shorter overall survival than those with lower expression levels. After computational analysis, we found that CTDSPL, a predicted target gene of miR-100-5p, also known as RBS3 (RB1 serine phosphatase chromosome 3), has a significant role in mediating the cell cycle progression. There is one binding sequence prediction in the 3^′^ UTR region of CTDSPL for binding to miR-100-5p, and these predicted sequences were conserved in humans (Fig. 3E). Further, western blot assay results confirmed that CTDSPL regulates the downstream target gene of miR-100-5p, where the level of CTDSPL was highly reduced in LoVo^OXR^ as compared with that in LoVo^WT^ cells (Fig. 3F). These results conclude that CTDSPL is a genuine target to miR-100-5p in CRC-resistant cells (Supplementary Fig. S2).

### 3.4 Relationship between CTDSPL Expression and Cell Cycle Progression in Chemoresistance-Associated CRC Cells

**Figure 4:**
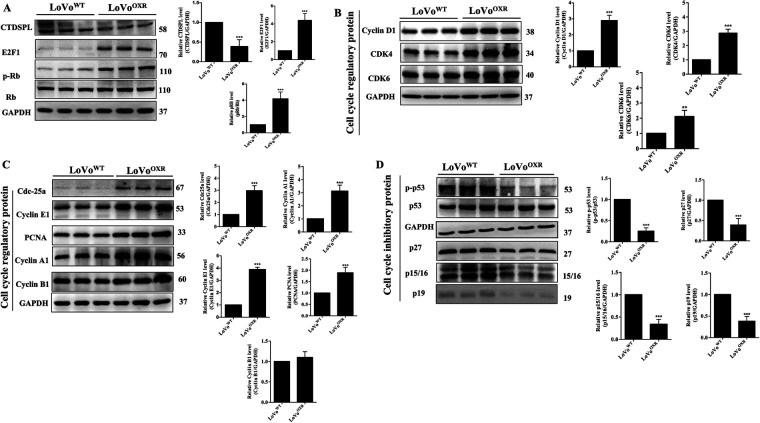
Lower CTDSPL expression is significantly associated with cell cycle alteration in OXR-LoVo cells. (**A**) Western blot analysis showing the expression levels of CTDSPL, pRB, RB and E2F1; (**B**,**C**) the cell cycle phase regulatory proteins (Cdc25a, cyclin E, cyclin A, cyclin D1, PCNA, Cdk4, Cdk6 and cyclin B1); (**D**) cell cycle inhibitory proteins (p-p53, p27, p15/16, p19) in parental and oxaliplatin-resistant LoVo cells. GAPDH was used as endogenous control. Quantification of data was carried out by ImageJ software 1.50 and is presented as the mean ± SD (*n* = 3). Significance ascribed as **p < 0.01, ***p < 0.001 vs. oxaliplatin-resistant LoVo cells.

miR-100-5p mimic down regulate CTDSPL expression, whereas miR-100-5p inhibition upregulates CTDSPL expression in LoVo^WT^ cells (Supplementary Fig. S3). According to our findings, miR-100-5p was overexpressed in oxaliplatin-resistant CRC cells, while CTDSPL expression was suppressed. Further study confirmed with western blot assay, which showed the decreased expression levels of CTDSPL along with an increased level of pRB and E2F1 were found in the overexpressed miR-100-5p LoVo^OXR^ cells than those in LoVo^WT^ cells, and the level of total RB protein remained the same in both cells (Fig. 4A). E2F1 functions as a master of the cell cycle regulator gene, which is involved in DNA replication, cell cycle progression, and cell proliferation in cancer. The different E2F1-target cell cycle regulatory genes were evaluated through a western blot assay to examine whether E2F1 may contribute to cell cycle progression in the LoVo^OXR^ cells. A significant increase in the expression levels of cyclin D1, Cdk4, Cdk6, Cdc25a, cyclin E1, PCNA, Cyclin A1, and, Cyclin B1 (Fig. 4B,C) but did not show increased expression levels of cell cycle inhibitory proteins p15/p16 INK4a, p27, p-p53, and p19 were observed in LoVo^OXR^ than in LoVo^WT^ cells (Fig. 4D). In addition, we analyzed the expression level of cyclin B1 which was unchanged between parental and LoVo^OXR^ cells (Fig. 4C). This study demonstrated a link between the expression of CTDSPL and cell cycle progression in CRC cells associated with chemoresistance.

### 3.5 Determination of the Upstream Target of miR-100-5p in Chemoresistant CRC Cells

**Figure 5:**
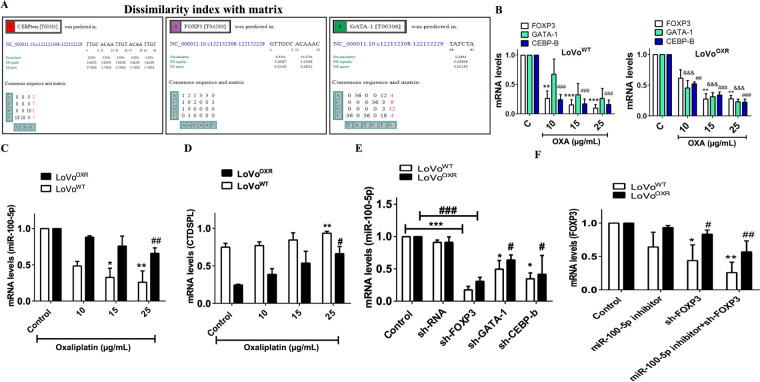
Determination of the upstream target of miR-100-5p in chemoresistance CRC cells. (**A**) Through computational-based (http://alggen.1si.upc.es/cgi-bin/promo_v3) with dissimilarity index 15% we were determined the upstream target of miR-100-5p in chemoresistance CRC cells are *GATA-1*, *CEBP-β*, and *FOXP3*. (**B**) mRNA levels of the upstream target (*GATA-1*, *CEBP-β*, and *FOXP3*) of miR-100-5p chemoresistance CRC cell after treated with oxaliplatin drug (**C**) miR-100-5p levels was decreased in colorectal cancer cells after oxaliplatin treatment. (**D**) CTDSPL in colorectal cancer cells is negatively linked to miR-100-5p. (**E**) sh-RNA approach and qPCR method were used to measure the expression of miR-100-5p after being treated with sh-RNA (sh-FOXP3, sh-GATA-1, sh-CEBPb) in LoVo^WT^ and LoVo^OXR^ CRC cells. Silencing of upstream target (*GATA-1*, *CEBP-β*, and *FOXP3*) affected miR-100-5p level in LoVo^WT^ and LoVo^OXR^. (**F**) Sh-FOXP3 suppressed the mRNA levels of FOXP3. All the RNA levels were measured by qPCR analysis. Values shown are mean ± standard deviation (*n* = 3). Significance ascribed as *p < 0.05, **p < 0.01, ***p < 0.001 represents the significance compared with control group LoVo^WT^ cells, whereas ^#^*p* < 0.05, *^##^p* < 0.01, *^###^p* < 0.001 represents the significance compared with control group oxaliplatin-resistant LoVo cells. ^&&&^*p* < 0.001.

We expanded our research into CTDSPL as a potential target of miR-100-5p in chemoresistant CRC cells. Next, we examined which gene regulates miR-100-5p as the upstream target through screening bioinformatics databases, such as (http://alggen.1si.upc.es/cgi-bin/promo_v3) to check if the factor predicted was within a 15% dissimilarity margin. We discovered three candidate upstream gene targets of miR-100-5p by comparing the dissimilarity index to the matrix: CCAAT enhancer-binding protein beta (C/EBP beta), GATA binding protein-1 (GATA-1), and forkhead box protein p3 (FOXP3) (Fig. 5A). Following our finding, the expression of mRNA levels of C/EBP beta and FOXP3 was significantly lower than GATA-1 in LoVo^WT^ CRC cell lines after treatment with a variable dose of oxaliplatin; Meanwhile the expression of FOXP3 was high in the LoVo^OXR^ CRC cells (Fig. 5B). We also discovered that after oxaliplatin treatment, the expression of miR-100-5p decreased (Fig. 5C), whereas CTDSPL expression increased (Fig. 5D). This finding indicates that FOXP3 might have been influenced and displayed as upstream target of miR-100-5p in chemotherapy-resistant CRC cells. Previous research has shown that FOXP3 promotes CRC cells’ proliferation, migration, and invasion by activating the AKT signaling pathway and regulating 15-lipoxygenase-1 expression, which serves as a promoter in basic transcription. Furthermore, we used the shRNA assay to determine that these three transcription factor genes influence miR-100-5p levels in the LoVo^WT^ and LoVo^OXR^ CRC cells. The results revealed that the expression of miR-100-5p with silencing FOXP3 (sh-FOXP3) was significantly lower compared with sh-GATA-1 and sh-CEBP-b in CRC cell lines (Fig. 5E). Further determination, miR-100-5p inhibitor and sh-FOXP3 were transfected into LoVo^WT^ and LoVo^OXR^ CRC cells by using an RT-PCR assay, which revealed that the expression of FOXP3 was effectively reduced (Fig. 5F). Thus, we believed that FOXP3 has influenced miR-100-5p regulation in the development of chemotherapy-resistant colorectal cancer.

### 3.6 FOXP3 Is the Upstream Target of miR-100-5p in Chemoresistance CRC Cells

**Figure 6:**
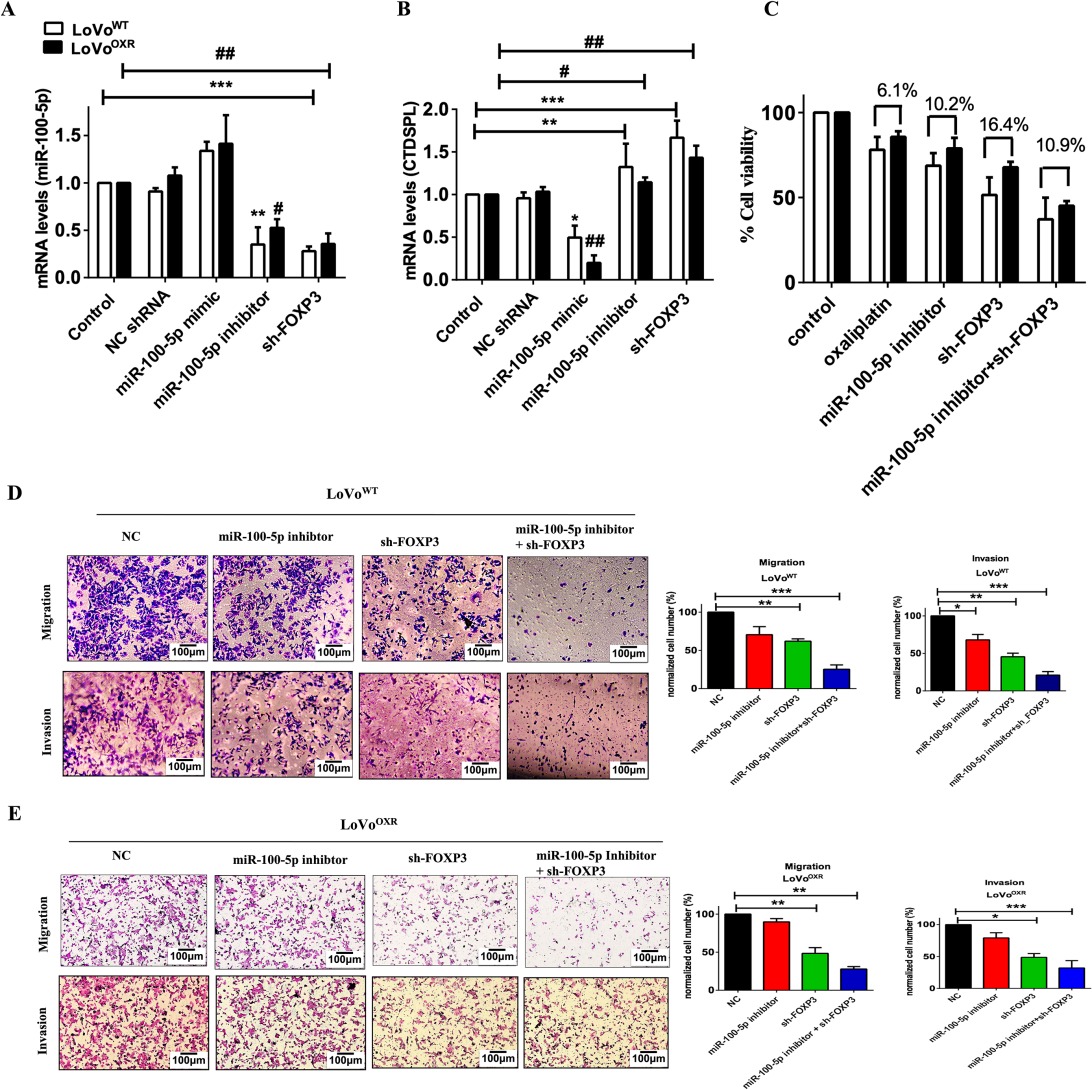
Forkhead box protein p3 (**FOXP3**) **is potential upstream target of miR-100-5p in chemoresistance CRC cells.** (**A**,**B**) LoVo^OXR^ and LoVo^WT^ CRC cells transfected instantaneously with siRNA FOXP3, mimic miR-100-5p and inhibitor miR-100-5p were determined by RT-PCR assays (**C**) MTT assays showed that transfection of either sh-FOXP3 and miR-100-5p inhibitor inhibited cell viability in LoVo^OXR^ and LoVo^WT^ CRC cells. (**D**) Transwell assay for migration and invasion ability of LoVo^WT^ CRC cells transfected with mimic miR-100-5p, sh-FOXP3 and both. (**E**) Transwell assay for migration and invasion ability of LoVo^OXR^ CRC cells transfected with mimic miR-100-5p, sh-FOXP3 and both. Transwell assays showed either miR-100-5p inhibitor or sh-FOXP3 suppressed migration and invasiveness in LoVo^OXR^ and LoVo^WT^ CRC cells. Significance ascribed as *p < 0.05, **p < 0.01, ***p < 0.001 represents the significance compared with control group LoVo^WT^ cells, whereas ^#^*p* < 0.05, *^##^p* < 0.01 represents the significance compared with control group vs. oxaliplatin-resistant LoVo cells.

FOXP3 was discovered to be highly expressed in chemoresistant colorectal cancer; we then investigated whether silencing FOXP3 affected miR-100-5p levels and CTDSPL expression. sh-FOXP3 was transfected into LoVo^WT^ and LoVo^OXR^ CRC cells. Interestingly, we revealed that miR-100-5p expression was down-regulated by silencing FOXP3 and a miR-100-5p inhibitor in LoVo^WT^ and LoVo^OXR^ CRC cells (Fig. 6A). Meanwhile, CTDSPL expression was up-regulated in chemoresistant CRC cells (Fig. 6B). These findings led us to believe that FOXP3 is the upstream target of miR-100-5p, which is over-expressed in the LoVo^OXR^ CRC cells. More importantly, CTDSPL, known as the downstream target of miR-100-5p, was also suppressed, resulting in an enhanced cell cycle in the chemoresistant CRC cells. In addition, MTT assays determined that either the silencing of *FOXP3* or the miR-100-5p inhibitor significantly inhibited the growth of oxaliplatin-resistant CRC cells. Importantly, the results show that inhibiting tumor growth in chemoresistant CRC cells with silencing gene target *FOXP3* and miR-100-5p inhibitor was more effective than chemotherapy drug alone (Fig. 6C). Silencing FOXP3 and the miR-100-5p inhibitor in LoVo^WT^ and LoVo^OXR^ CRC cells also significantly reduced cell migration ability (Fig. 6D), which was also demonstrated by decreased cell invasiveness (Fig. 6E). The identification of FOXP3 as an upstream target of miR-100-5p completes our understanding of the role of miR-100-5p in the cell cycle regulation of chemoresistant CRC cell lines.

### 3.7 The Role of miR-100-5p in Chemoresistant CRC Cells

**Figure 7:**
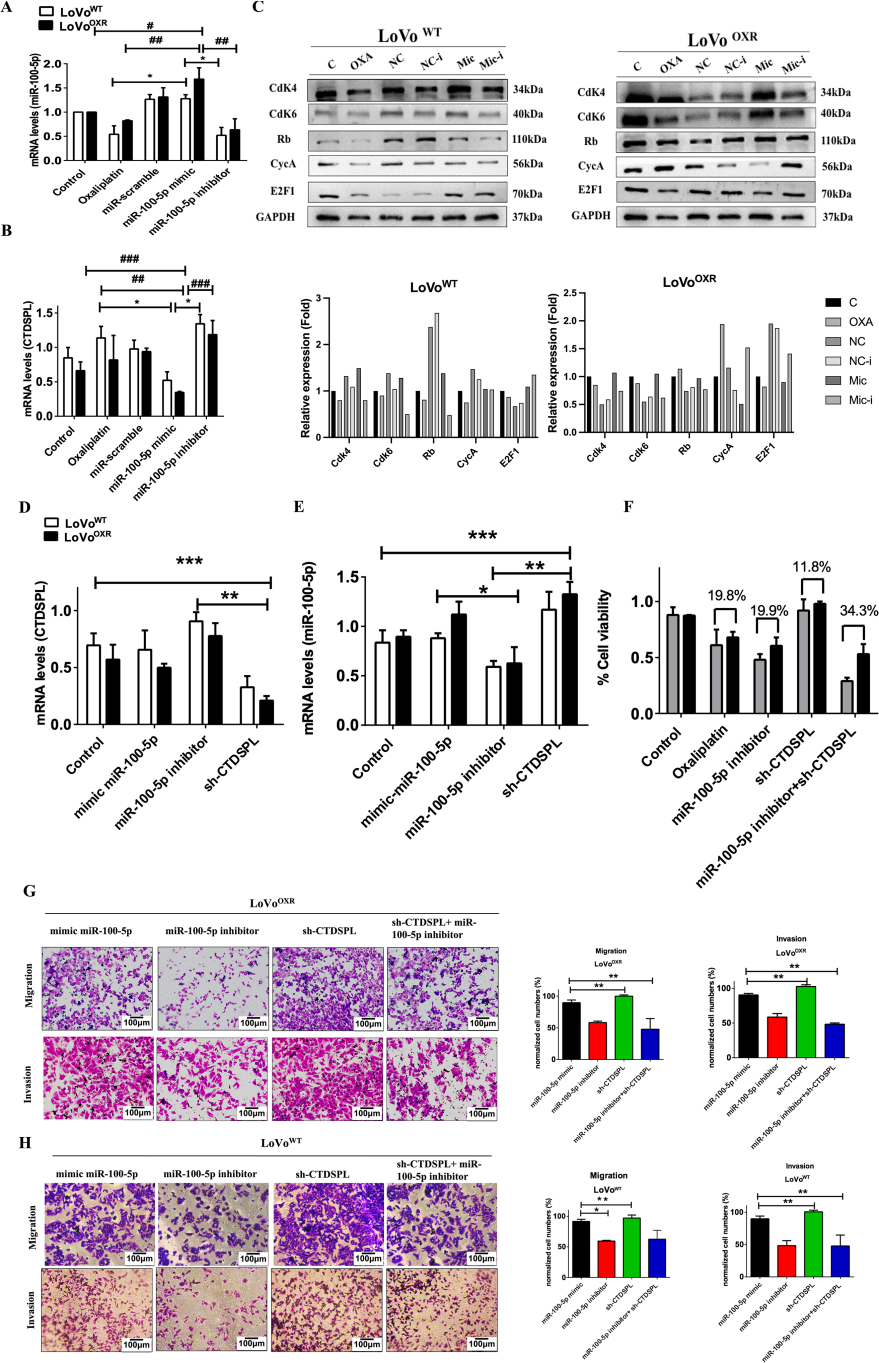
miR-100-5p targets CTDSPL which modulates the cell cycle—mediated chemoresistance in CRC cells. (**A**) Following miR-100-5p inhibitor action, suppressed the level of miR-100-5p mRNA in LoVo^OXR^ and LoVo^WT^ CRC cells, and (**B**) The inhibition of miR-100-5p increased the level of *CTDSPL* expression, (**C**) Western blot assays showed transfection of miR-100-5p inhibitor also affected the protein level of cell cycle regulator and proliferation marker (CDK4, CDK6, E2F1 and Rb), which were decreased in the chemoresistance colorectal cell. (**D**,**E**) LoVo^OXR^ and LoVo^WT^ CRC cells transfected instantaneously with siRNA CTDSPL, mimic miR-100-5p and inhibitor miR-100-5p were determined by RT-PCR assays. (**F**) Silencing *CTDSPL* transfected in LoVo^OXR^ and LoVo^WT^ CRC cells lead to recovery of cell viability compared with miR-100-5p inhibitor transfection. (**G**) Transwell assay for migration and invasion ability of LoVo^WT^ CRC cells transfected with miR-100-5p mimic, miR-100-5p inhibitor, sh-CTDSPL and miR-100-5p inhibitor+sh-CTDSPL. (**H**) Transwell assay for migration and invasion ability of LoVo^OXR^ CRC cells transfected with miR-100-5p mimic, miR-100-5p inhibitor, sh-CTDSPL and miR-100-5p inhibitor+sh-CTDSPL. Transwell assays showed either mimic miR-100-5p or sh-CTDSPL enhanced migration or invasiveness in LoVo^OXR^ and LoVo^WT^ CRC cells. Using the qPCR method all mRNA levels were assessed. GAPDH was used as endogenous control. Quantification of western blot data was carried out by ImageJ software 1.50 and is presented as the mean ± SD (*n* = 3). Significance ascribed as *p < 0.05, **p < 0.01, ***p < 0.001 represents the significance compared with control group LoVo^WT^ cells vs. oxaliplatin-resistant LoVo cells, whereas ^#^*p* < 0.05, *^##^p* < 0.01, *^###^p* < 0.001 represents the significance compared with control group vs. oxaliplatin-resistant LoVo cells.

Following the findings, we discovered that CTDSPL, a downstream target of miR-100-5p and FOXP3, the upstream target of miR-100-5p are associated with cell cycle progression and the proliferation rate of oxaliplatin-drug resistance in CRC cells. In order to determine the biological function relevance between CTDSPL and miR-100-5p, we asked whether restoration of miR-100-5p expression by transfection of miRNA mimics (referred to simply as miR-100-5p mimic) could rescue tumor development mediated by suppressing CTDSPL in LoVo^WT^ and LoVo^OXR^ CRC cells. RT-PCR assays showed that transfection of mimics effectively restored the expression level of miR-100 5p compared with oxaliplatin treatment and miR-100-5p inhibitors in LoVo^WT^ and LoVo^OXR^ CRC cells (Fig. 7A). Meanwhile, CTDSPL expression in the mimic miR-100-5p decreased significantly and was restored after treatment with a miR-100-5p inhibitor in chemoresistant colorectal cancer (Fig. 7B). Importantly, the miR-100-5p inhibitor revealed suppressed cell cycle regulation and proliferation rate, as shown by the fact that Cdk4, Cdk6, Rb, CycA, and E2F1 expression levels were down-regulated in the LoVo^OXR^ cells. The transfection of a mimic of miR-100-5p effectively restored the level of protein cell cycle regulation in LoVo^WT^ and LoVo^OXR^ CRC cells (Fig. 7C). Silencing CTDSPL as further validation to determine whether CTDSPL is a downstream target of miR-100-5p. RT-PCR assays revealed that the level of miR-100-5p increased significantly after CTDSPL silencing compared to control in chemoresistance CRC cells (Fig. 7E), whereas CTDSPL levels decreased significantly when compared to control and miR-100-5p inhibitor (Fig. 7D). Moreover, silencing CTDSPL in LoVo^WT^ and LoVo^OXR^ CRC cells leads to aggressive of cell growth compared than control (Fig. 7F). We also verified that inhibition expression of miR-100-5p inhibited migration ability and invasiveness in chemoresistance CRC cells, whereas silencing CTDSPL in the oxaliplatin chemoresistance CRC cells resulted in recovery of cell migration and invasion ability (Fig. 7G,H). We confidently stated that in chemoresistant colorectal cancer, miR-100 5p over-expression down-regulated CTDSPL expression and promoted pRB/E2F1 cell cycle regulator, which was reversed when miR-100 5p was inhibited. These results suggest that miR-100-5p expression alters cell cycle progression in oxaliplatin-resistant CRC cells by targeting the CTDSPL/pRB/E2F1 pathway. Taken together, these findings lead us to conclude that miR-100-5p plays an oncogenic role in the development of chemoresistance in CRC cells.

## 4 Discussion

We revealed that the miR-100-5p gene plays an oncogenic role in the development of chemoresistance in CRC cells, which is associated with altered cell cycle progression, reduction of apoptosis, and promotion of cell proliferation via regulation of the CTDSPL/pRB/E2F1-mediated signaling pathway. Decreased expression of CTDSPL along with an increased expression of pRB and E2F1 observed in LoVo^OXR^ cells compared to LoVo^WT^ cells, and this was associated with a high proliferation rate. Thus, CTDSPL expression was negatively correlated with the expression of miR-100-5p in drug-resistant CRC cells. As a result, our findings conclude that miR-100-5p may play an important role in regulating cell cycle-mediated chemoresistance in CRC cells.

Previous reports had reported that miR-100-5p expression was associated with chemotherapeutic resistance to cancer [26,27]. miR-100-5p promoted prostate cancer proliferation, and silencing the miR-100-5p gene enhanced apoptosis and reversed castration-induced resistance in cancer cells [28]. Clinical data from TGCA analysis from our present study also confirms the strong correlation of miR-100-5p expression with CRC mortality through CTDSPL. The downregulation of miR-100-5p expression significantly decreased non-small cell lung cancer (NSCLC) proliferation, ability to colony-form, and significantly enhanced sensitivity of NSCLC cells to the first line of chemotherapeutic drugs (crizotinib, lorlatinib, and cisplatin) [28]. The silencing of miR-100-5p was also observed to increase the sensitivity or resistance of epithelial ovarian cancer to cisplatin [29]. In addition, upregulation of miR-100-5p decreased the proliferation, migration, and colony-forming ability of numerous cancer cells, including prostate cancer, gastric cancer, endometrial cancer, chordoma cancer, and CRC [15,30,31]. In the present study, we observed that the upregulation of miR-100-5p expression analysis, as determined by microarray and qRT-PCR assays, significantly promoted cell proliferation and viability in chemoresistance CRC cells more than that in the LoVo^WT^ cell. Further, in CRC cells, oxaliplatin-induced apoptosis was prominently decreased compared to LoVo^WT^ CRC cells, and its sensitivity was significantly increased in LoVo^WT^ CRC cells. Therefore, increased expression of miR-100-5p might be associated with chemotherapy resistance in CRC cells.

**Figure 8:**
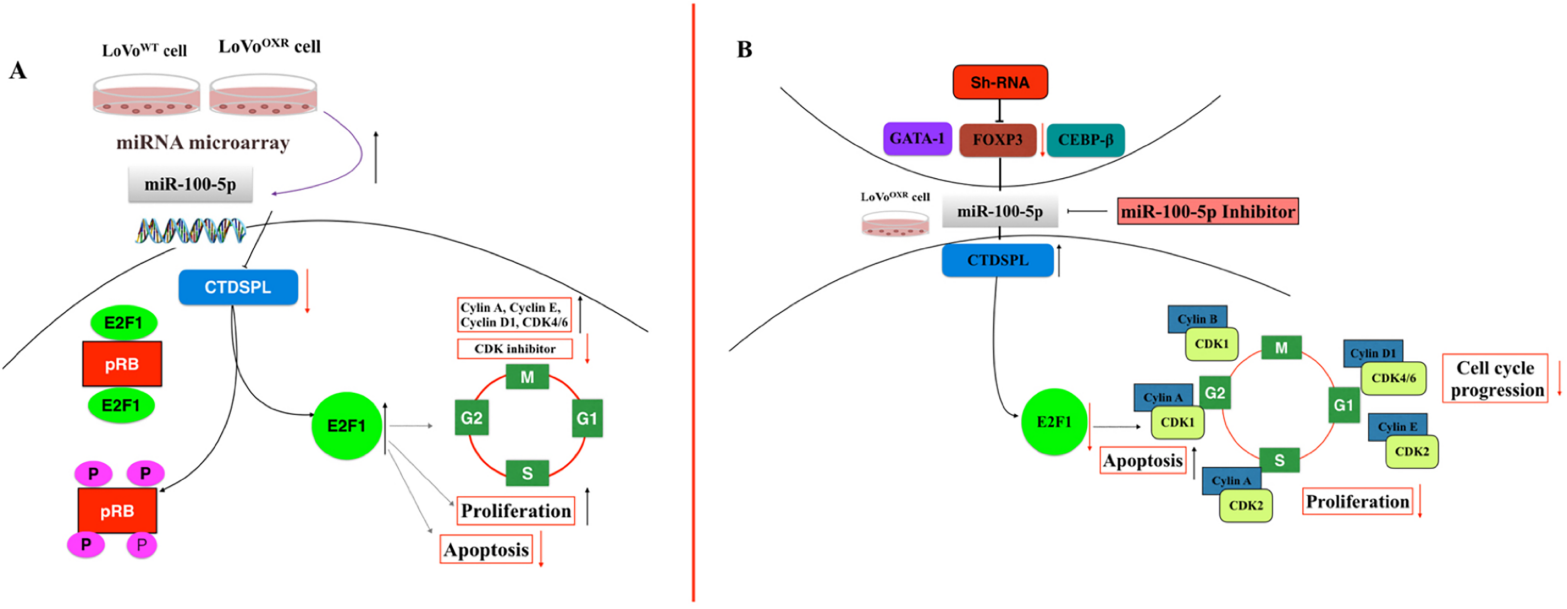
Proposed mechanism of miR—100-5p enhanced cell cycle—mediated chemoresistance achieved modulation of CTDSPL/pRB/E2F1 signaling pathway in CRC. (**A**) The miR-100-5p level is highly expressed in LoVo^OXR^; and negatively regulates CTDSPL level in the downstream pathway which was enhancing the cell cycle regulator and proliferation rate in chemoresistance colorectal cancer. (**B**) Reversed result, the inhibition of miR-100-5p highly regulates the expression of CTDSPL which promotes apoptosis in chemoresistance CRC cells. Additionally, FOXP3 is the upstream target of miR-100-5p in chemoresistance colorectal cancer cells and also an important genes target that involved in the development chemoresistance CRC cell lines. The red arrows represent decreasing marks, and the black arrows represent increasing marks. Abb: CTDSPL, C-terminal domain small phosphatase-like; E2F1, E2F transcription factor 1; pRB, Phosphorylated retinoblastoma protein; CDK inhibitor, Cyclin-Dependent Kinase inhibitor; CDK1, Cyclin-Dependent Kinase 1; CDK2, Cyclin-Dependent Kinase 1; CDK4/6, Cyclin-Dependent Kinase 4 and 6.

Cell cycle regulatory genes can deregulate proliferation, cell growth, and cell cycle progression, resulting in various pathological conditions, including human malignancy [22,32]. In CRC cells, the miRNA miR-486-5p inhibited CRC cell proliferation, invasion, and migration and also induced cell cycle arrest at the G1 phase by targeting CDK4 [33]. He et al. [34] reported that miR-218-induced apoptosis inhibited cell proliferation and caused cell cycle arrest at the G2 phase in colon cancer cells. However, Li et al. [35] reported that miR-18b significantly promoted CRC cell proliferation, metastasis, and cell cycle progression by targeting CDKN2B. Our present study showed that the cell cycle phase progression was increased in chemoresistance CRC cells than in parental CRC cells. Bioinformatics analysis and western blot assay results confirmed that CTDSPL was the predicted target of miR-100-5p. The CTDSPL gene, previously known as RBPS3 or HYA22, is a phosphatase-like-tumor suppressor gene located at 3p21.3 and belongs to the small C-terminal domain phosphatase family, which removes a phosphate group from the serine of Rb1 on ser-807 and ser-811. CTDSPL modulates the pRB/E2F1 mediated signaling cascade and induces cell cycle arrest at the G1/S boundary [22,23]. This association of CTDSPL levels and the increase in pRB and E2F1 levels in oxaliplatin-resistant CRC cells compared with parental CRC cells (Fig. 8A) are consistent with earlier studies, wherein downregulation of CTDSPL levels promoted cell proliferation, myeloid differentiation, and cell cycle progression (S-phase progression) in miR-100-5p and miR-181b expressing uveal melanoma and acute myeloid leukemia cells, respectively [20,30]. E2F1, a critical downstream binding protein of tumor suppressor protein Rb, can function as a master of the cell cycle regulator gene through the transcriptional regulation of dozens of genes involved in DNA replication and cell cycle progression and also plays a critical role in cancer cell proliferation. The ‘Rb’ pathway has a crucial role in cell physiology and different pathologies, including malignant transformation via different molecular mechanisms, through meditation of G1-phase cycle progression and the G1/S-phase transition [36,37]. In the present study, we found that upregulation of pRB expression enhanced the disruption of the pRB/E2F1 complex and, subsequently, the release of E2F1, which could further increase the cell cycle progression (G1-S and S-phase transition) and cell proliferation in chemoresistance CRC cells. Emerging evidence recommends that the expression of E2F1 was high in various types of human malignancies, and it was positively associated with high-grade tumors and poor prognosis conditions [38,39]. Moreover, cell cycle progression was positively associated with the overexpression of E2F1 in different cancer tissues [40] suggesting that E2F1 expression might regulate cell cycle development and progression of tumors. Chang et al. [41] reported that the activation of E2F1 by FGF9 was involved in the cell cycle progression and proliferation of mouse Leydig tumor cells.

Baldini et al. [42] showed that E2F1-overexpression was positively correlated with increased cyclin A and Ki67 expression and, as a result, reduced disease-free survival in node-negative breast cancer patients. Further, earlier studies illustrated that the activation of E2F1 leads to the transcription of genes encoding cell cycle progression and DNA synthesis-related proteins such as cyclin E, cyclin D, cyclin A, Cdk2, Cdk4/6, and cdc25 [40]. The cell cycle is essential in regulating cell growth. The complex of cyclin and Cdks proteins can promote cell cycle progression from one phase to another, and their deregulation during the cell cycle transit normal cells into malignant cells [43]. Levels of protein markers in the G1 phase, S-phase, and S/G2 phase are cyclin D1, cyclin E1, Cdc25a, Cdk4, and Cdk6 in the G1 phase; the cyclin E1, Cdk2, and PCNA levels for the S-phase, and the cyclin A1 and Cdk2 levels the for S/G2 phase were increased, whereas the level of cyclin B1 for the G2/M phase was not changed in LoVo^OXR^ CRC cells than that in the LoVo^WT^ cells. Next, we examined the status of cell cycle inhibitor proteins in LoVo^WT^ and chemoresistant CRC cells. p53 is a well-known cell cycle inhibitory protein and functions as a tumor suppressor that can downstream regulate p21 and p27 proteins to induce cell cycle arrest through interaction with the cyclin/Cdk complex. Here, we observed that the expressions of p15, p16, and p19 for the G1-phase and p-p53 and p27 for the G1-S phase and S-phase were reduced in oxaliplatin-resistant CRC cells compared to those in the parental CRC cells, indicating that the p53 and other cell cycle inhibition pathways do not play any role in suppressing chemoresistant CRC cell proliferation. Studies have shown that cell proliferation in numerous tumor tissues is due to the inactivation of the cell cycle inhibition pathway and activation of the pRB-E2F1 mediated pathway. In general (non-proliferating context), Rb is maintained in a d dephosphorylated state by phosphatase-like family proteins and interacts with DNA-bound E2F1, inhibiting its transcription function necessary for the G1-S and S-phase transition. The pRB-mediated pathway is functionally inactive in most cancer cells due to the deregulation of E2F1 activity, leading to uncontrolled proliferation. Our data also showed that expression of standard cell proliferation markers Ki67, p-AKT, and p-ERK1/2 was increased in LoVo^OXR^ CRC cells compared with that in LoVo^WT^ cells, indicating the pRB/E2F1-mediated pathway plays a significant role in CRC cells proliferation. Many studies have shown that E2F1 dependent cells proliferation was found to be mediated by up-regulation of EGFR expression and activation of cytoplasmic Ras/mitogen-activated protein kinase (MAPK)/extracellular signal-regulated kinase (ERK) and PI3K/AKT signaling cascades as well as transactivation of Ki67 *in vivo* and *in vitro* [41,42,44]. Thus, our results were parallel to other previous studies.

The miR-100-5p inhibitor was used to confirm that the CTDSPL expression was upregulated, which led to apoptotic and deactivated cell proliferation. To extend our findings, we checked the upstream target of miR-100-5p. FOXP3 has a vital role in the development of CRC. Further, the qRT-PCR results showed that FOXP3 was significantly upregulated in miR-100-5p, which suppressed CTDSPL expression. In contrast, the expression level of miR-100-5p was decreased treated with sh-FOXP3 (Fig. 8B). Therefore, we concluded that FOXP3 is a mediator of miR-100-5p regulation in chemoresistance CRC cell lines, supported by previous reports that FOXP3 expression in CRC cells correlates with disease progression in CRC patients [45], also as a marker of CD4^+^CD25^+^ regulatory T cells which is determinant of their immunosuppressive functions and shown limit anti-tumor immune responses during tumor progression. From a therapeutic perspective, modulating the miR-100-5p/CTDSPL axis may represent a promising strategy to overcome oxaliplatin resistance in CRC. Inhibitors of miR-100-5p or small-molecule activators that restore CTDSPL activity could potentially reestablish cell cycle control and sensitize resistant cells to chemotherapy. However, *in vivo* and patient-based studies are needed to validate this pathway and evaluate its feasibility as a therapeutic target. Future studies will focus on testing these interventions in xenograft and patient-derived CRC models to confirm their clinical relevance.

Although the present study revealed the role of FOXP3/miR-100-5p/CTDSPL regulatory axis in oxaliplatin resistant CRC, several limitations are present, which should be acknowledged. The experiments were performed in a single CRC cell line model (LoVo^WT^ and LoVo^OXR^), which may not fully represent the genetic diversity of colorectal tumors. The chemoresistance mechanisms can differ across CRC cell lines with distinct genetic and molecular backgrounds (such as HCT116, SW480, and DLD1). The absence of clonogenic and *in vivo* studies limits the translational extrapolation of the findings. Gain or loss of function rescue assays are needed to establish the functional role of CTDSPL in oxaliplatin resistance. Finally, short term viability and apoptosis assays may not fully reveal the long term adaptation of resistant cells. Future studies incorporating additional CRC models, *in vivo* xenografts, and patient-derived samples are warranted to confirm the clinical relevance of this pathway.

## 5 Conclusion

In summary, we found that miR-100-5p plays an oncogenic role in oxaliplatin-resistant colorectal cancer. In LoVo^OXR^ cells, cell proliferation markers are upregulated along with miR-100-5p. Further, we reported that FOXP3 was the upstream target of miR-100-5p, which mediated the regulation of miR-100-5p expression in chemoresistant CRC cells, thereby influencing the downstream target of miR-100-5p, CTDSPL expression in CRC cells. Further validation across multiple CRC models, *in vivo* models, and patient cohorts is necessary to establish the translational significance of this pathway.

## Supplementary Materials



## Data Availability

All data generated or analyzed during this study are included in this article. More data are available from the corresponding authors for reasonable request.

## Supplementary Materials

The supplementary material is available online at https://www.techscience.com/doi/10.32604/or.2026.073080/s1.
