# Supplementary material for: miR-100-5p Enhances Cell Cycle-Mediated Chemoresistance by Modulating the CTDSPL/pRB/E2F1 Signaling Pathway in Oxaliplatin-Resistant Colorectal Cancer Cells
Source: Oncol Res. 2026 Mar 23;34(4):17. doi: 10.32604/or.2026.073080 (PMC13040292; doi:10.32604/or.2026.073080)
Supplement: Supplementary file 1 [file OncolRes-34-73080-s001.docx]

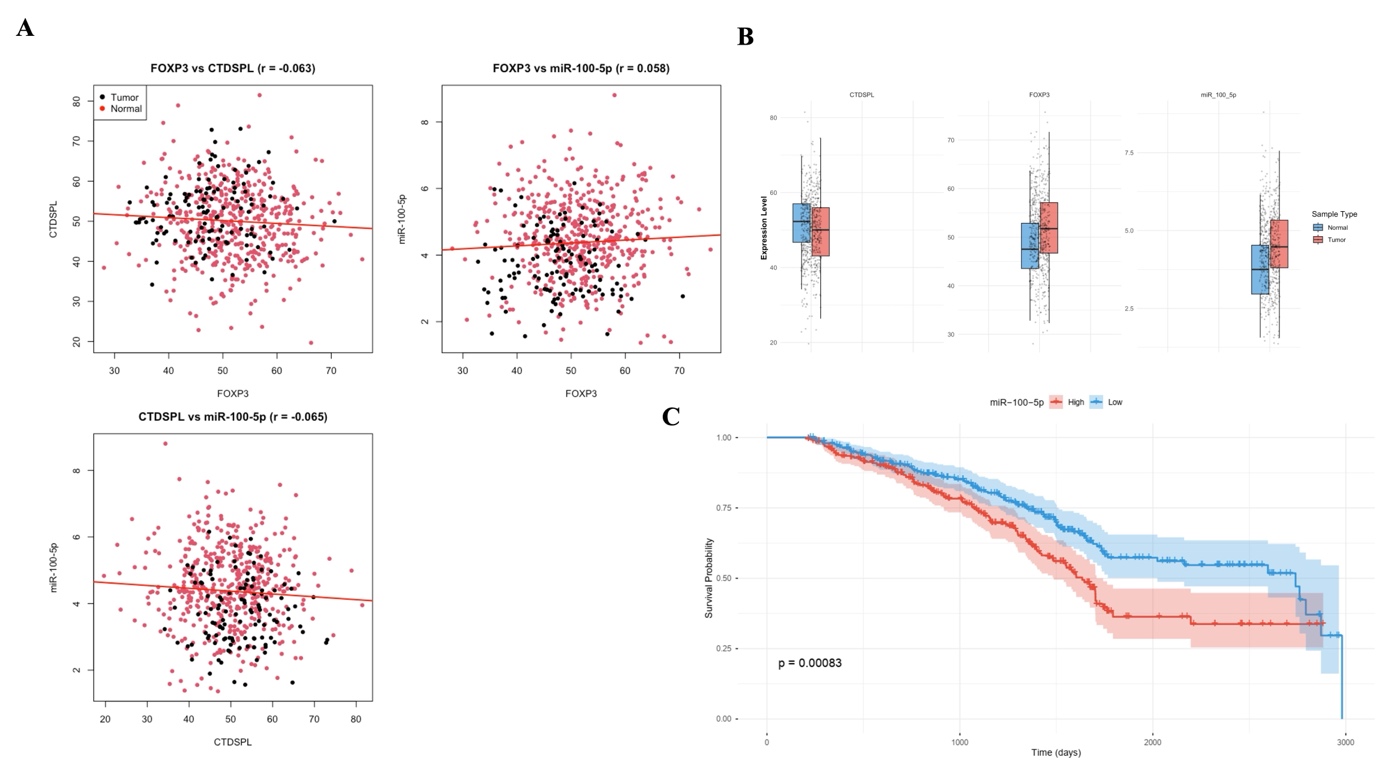


**Supplementary Figure S1.** miR-100-5p expression correlation analysis using The Cancer Genome Atlas (TGCA) dataset analysis. (A) Gene correlation analysis of miR-100-5p, forkhead box P3 (FOXP3), and C-terminal domain small phosphatase-like (CTDSPL) expression in normal and colon cancer patient’s sample. (B) Expression patten of miR-100-5p, FOXP3, and CTDSPL in normal and CRC patients. (C) Kaplan−Meier survival analysis showing expression levels of miR-100-5p had significantly shorter overall survival than those with lower expression levels.

**
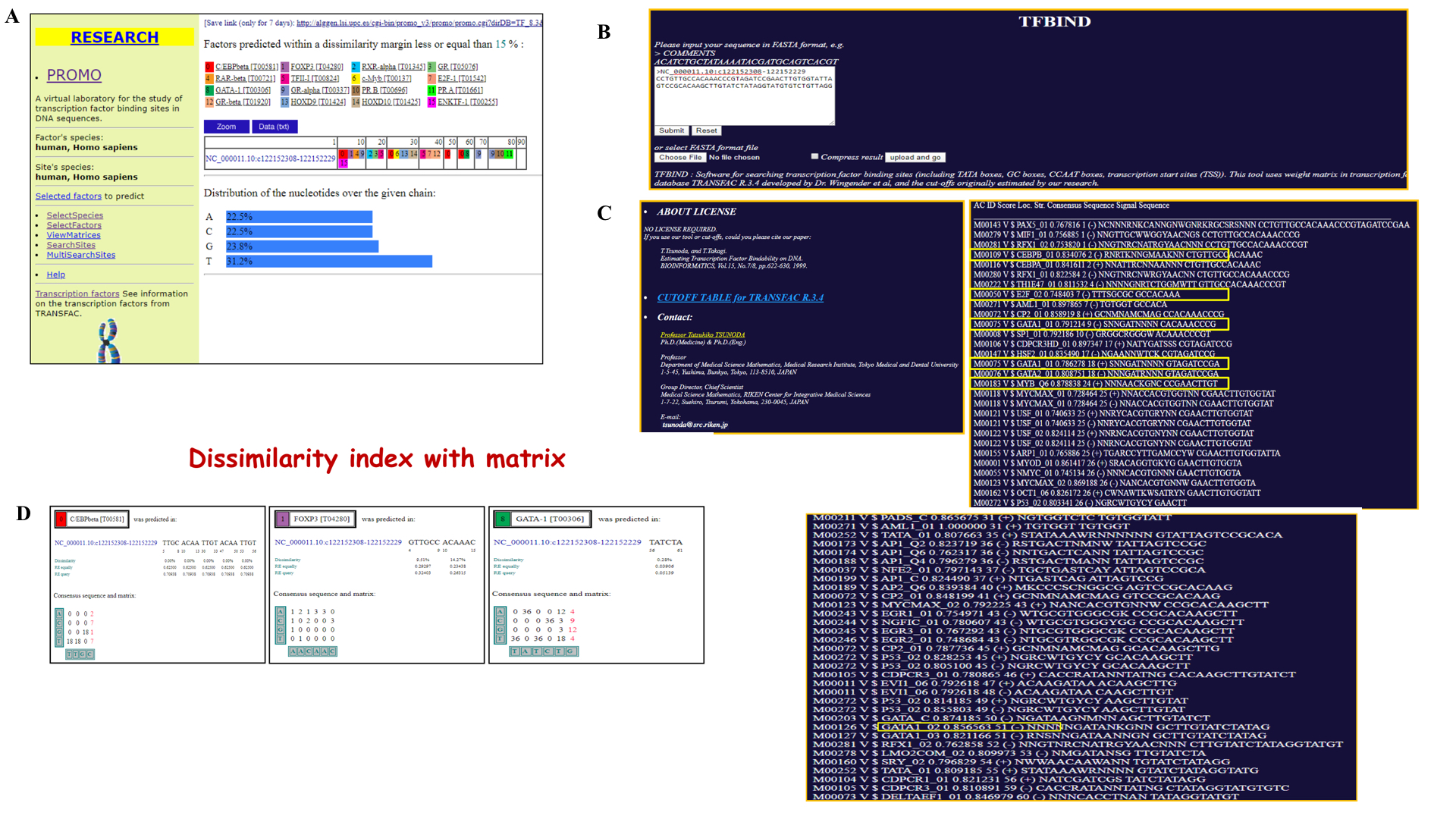
**

**Supplementary Figure S2.** Prediction and analysis of transcription factor binding sites using bioinformatic Profiler of Multi-Omic data (PROMO) and Transcription Factor Binding Sites (TFBIND) tools. The prediction of transcription factor binding sites (TFBS) was performed using the PROMO and TFBIND online tools (http://alggen.1si.upc.es/cgi-bin/promo_v3). (A) PROMO identified putative transcription factors binding to the given DNA sequences with a dissimilarity margin < 15%. The nucleotide distribution across the input sequence (A,C,G,T) is shown in the bar graph format. (B) TFBIND analysis is provided detailed matches between the input sequence transcription factor binding motifs from TRANSFAC database, showing factor name, binding position, strand orientation and dissimilarity score. (C) Highlighted results represent the most probable binding sites with the lowest dissimilarity index values. (D) The dissimilarity index matrix comparison between consensus and predicted sequences, showing matches binding for FOXP3, GATA-1 and C/EBPβ binding motifs


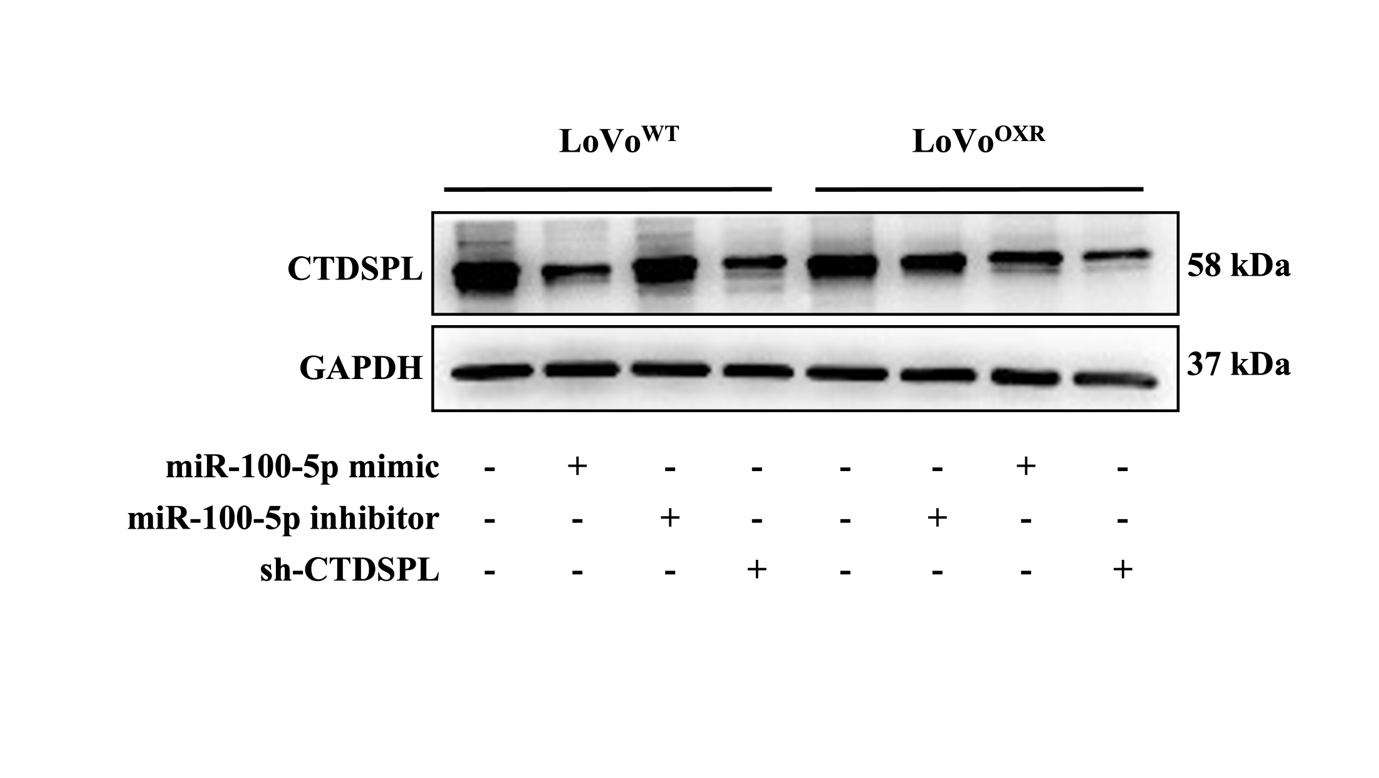


**Supplementary Figure S3.** Western blot showing the expression pattern of CTDSPL in LoVo^WT^ and LoVo^OXR^ by mimicking and inhibiting miR-100-5p.
